# Study on sewage characteristics in rural China and pollutants removal performance of biologically enhanced internal circulation treatment system

**DOI:** 10.1038/s41598-023-45085-4

**Published:** 2023-10-23

**Authors:** Yi Rong, Hongjun Tang, Yang Zhang, Yingying Sun, Zhe Liu

**Affiliations:** 1 Institute of Land Engineering and Technology, Shaanxi Provincial Land Engineering Construction Group Co., Ltd., Xi’an, Shaanxi China; 2 Shaanxi Provincial Land Engineering Construction Group Co., Ltd., Xi’an, Shaanxi China

**Keywords:** Environmental biotechnology, Sustainability, Environmental impact

## Abstract

With rapid economic development and urbanization in China, rural wastewater treatment has become an important issue. This study investigated 63 rural sewage treatment stations in northern, central and southern Shaanxi, China for a 1-year period, 2021 to 2022. The main purpose of the research was to investigate the quality and discharge characteristics of rural sewage, along with current problems in rural wastewater treatment, in order to provide evidence for the optimal construction and operation of rural sewage treatment stations. We found that the biodegradability of rural wastewater is adequate, and BOD_5_/COD ratio in sewage was 0.4, which is suitable for biological treatment. It has obvious intermittent flow cut-off characteristics, and the range of cut-off duration of sewage was 6–16 h/d, which leads to poor pollutant removal efficiency (COD: 50.0 ± 29.2%, NH_4_^+^-N: 46.0 ± 26.1%, TN: 38.5 ± 24.9% and TP: 38.3 ± 23.8%) in sewage treatment stations. In response to the above characteristics, the rural sewage biologically enhanced internal circulation treatment (BEICT) system was constructed. After 97 days of operation, the system has a stable removal effect on TN and TP with an average removal rate of 77.42% and 89.69%, respectively, under the condition of influent interruption for 12 h per day. The activated sludge of system maintained good activity and stable sedimentation performance during the whole experiment, with MLVSS/MLSS and SVI of 0.72 and 128 mL/g, respectively. This study can provide the basis and technical support for the accurate design of rural sewage treatment facilities, and has important significance for guiding the treatment of rural domestic sewage in China.

## Introduction

With the rapid development of China's economy, the living conditions in China's rural areas has been greatly improved. The amount of rural sewage has increased year by year. According to the bulletin of the second national survey of pollution sources^1^, the total amount of water pollutants discharged in rural areas accounts for almost 40% of the entire Chinese emissions, including chemical oxygen demand (COD), ammonia nitrogen (NH_4_^+^-N), total nitrogen (TN) and total phosphorus (TP) accounted for 51%, 35%, 30% and 39% of the total emission in the country, respectively. Only 6% of rural areas had wastewater treatment systems by the end of 2010^2^, increasing to 19% by the end of 2018^3^. China's rural areas still discharge a large amount of untreated sewage every year. This seriously hinders the process of improving the living environment in rural China. Improving rural living environment and rural sewage treatment capacity has gradually become an important development direction in environmental protection field^4,5^.

With the increase of investment in rural infrastructure by the Chinese government, a large number of sewage treatment facilities have been built in rural areas^6^. Due to the lack of detailed research on the characteristics of rural sewage discharges, most established rural sewage treatment facilities cannot operate efficiently and stably. This not only caused a waste of funds, but also brought a bad impact on the rural water environment.

Due to the uneven quality of sewage pipeline construction in rural areas, a large amount of sewage seeps into the ground or evaporates during collection^7^. China's rural residents live more dispersed and use less water, so the sewage discharge is often difficult to reach the design value of sewage treatment facilities, resulting in most sewage treatment facilities are often operating without influent^7–9^. Rural domestic sewage flows in China have significant fluctuations coupled with intermittent breaks, or discontinuities, in flow and this causes real problems that can not be ignored in the processing of rural sewage.

Domestic sewage treatment technologies in rural China are mainly based on ecological treatment, biological treatment or a combination of ecological and biological treatment^2^. With the rise of China's manufacturing industry, integrated bioreactors with anaerobic/anoxic/oxic (A^2^/O), anoxic/oxic (A/O) and membrane bioreactor (MBR) processes offer good alternatives in order to meet environmental requirements in China's rural areas^2,5,8,10^. These integrated bioreactors are often unable to operate efficiently and consistently in rural areas. This is closely related to the quality and quantity of sewage in rural areas of China. In order to research and develop sewage treatment technologies that can cope with the typical characteristics of rural wastewater in China, basic study about wastewater quality and quantities in Chinese rural areas is needed. Several studies have reported the characteristics of rural sewage discharge in some areas of China, most of them were based on source measurement, the quality and quantity of rural sewage transported through the rural sewage network to the sewage treatment facilities have not been reported^11–13^. Most studies only focused on the fluctuation of rural sewage quantity, but the discontinuous discharge characteristics caused by the fluctuation of rural sewage have not received attention, and the impact of such discharge characteristics on rural sewage treatment facilities is still unclear.

With the rapid construction of rural sewage treatment plants in China, residual sludge, as the main by-product of sewage treatment process, is being produced in large quantities and has become a new type of organic solid waste in rural environment^14^. It is urgent to develop a rural sewage treatment model that can improve the efficiency of pollutant removal and reduce the yield of residual sludge. The increase of residual sludge production in rural areas of China brings challenges and opportunities. Numerous studies have shown that a large amount of short-chain volatile fatty acids (VFAs, including acetic acid, propionic acid, butyrate, etc.) can be produced by anaerobic fermentation of waste activated sludge under certain conditions^15–17^. The fermentation broth containing VFAs can be used as a carbon source to improve the nitrogen and phosphorus removal efficiency of biological nutrient removal (BNR) system^18,19^. This makes it possible for the BNR system to be self-sufficient in its carbon sources. It has not been reported how to integrate anaerobic sludge fermentation with BNR system effectively and apply it to decentralized and small-scale rural sewage treatment. How to efficiently utilize the fermentation carbon source and make the BNR system run continuously and stably under the intermittent discharge of rural sewage is also worth further research.

This study was conducted in the Shaanxi Province, China, which is a typical representative of the economically comparatively developed areas in northwest China. There are 63 rural sewage treatment plants in Shaanxi Province were investigated for 1 year, aiming to investigate the sewage discharge characteristic in rural aeras and the pollutant removal performance of the sewage treatment stations. We also attempt to clarify the impact of rural sewage discontinuous discharge on sewage treatment facilities through correlation analysis, and proposed the biologically enhanced internal circulation treatment system (BEICT) for rural sewage, in order to solve the problem of stable treatment of sludge and sewage simultaneously under the characteristics of rural sewage discontinuous discharge. A pilot-scale BEICT bioreactor was constructed and a long-term field operation study was carried out. This study can provide the basis and technical support for the accurate design of rural sewage treatment facilities, and has important significance for guiding the treatment of rural domestic sewage in China.

## Results and discussion

### Study on rural sewage quality

**Figure 1:**
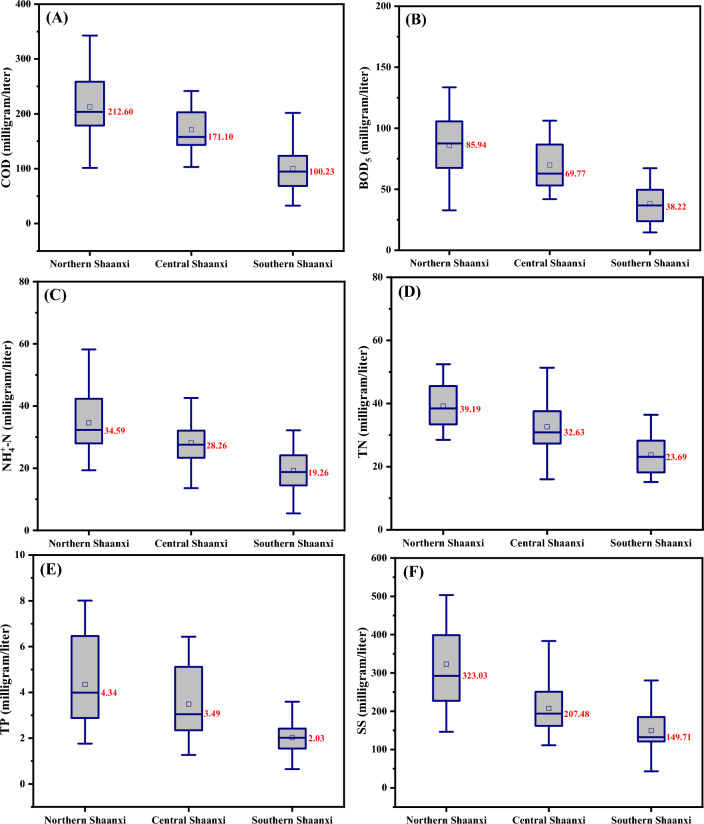
Concentration of COD (**A**), BOD_5_ (**B**), NH_4_^+^-N (**C**), TN (**D**), TP (**E**) and SS (**F**) in rural sewage in Shaanxi Province, China.

The influent pollutants concentrations of the 63 rural sewage treatment stations are shown in Fig. 1. The average influent concentrations over the year of COD, BOD_5_, NH_4_^+^-N, TN, TP and SS of rural sewage treatment stations in northern Shaanxi were 212.60, 85.94, 34.59, 39.19, 4.34 and 323.03 mg/L respectively. The average influent concentrations of COD, BOD_5_, NH_4_^+^-N, TN, TP and SS in central Shaanxi were 171.10, 69.77, 28.26, 32.63, 3.49 and 207.48 mg/L, and the average influent concentrations in southern Shaanxi were 100.23, 38.22, 19.26, 23.69, 2.03 and 149.71 mg/L. The comparison of rural sewage quality in Shaanxi Province with that in other districts of China is shown in Table 2 of the Supplementary Material. The concentration of pollutants in rural sewage in Shaanxi Province is obviously lower than that in Jiangsu, Guangdong and other economically developed areas in China, which is mainly related to the relatively backward network construction and residents' living habits in rural Shaanxi Province^13^.

Northern Shaanxi is located in the arid and semi-arid areas of northwest China, where serious soil and water loss, barren soil and a generally poor ecological environment occurs. This leads to high sand content in surface drainage and rural sewage. Due to the relatively under developed economy in this region, the quality of sewage pipeline planning and construction is low, resulting river water and industrial sewage entering pipelines prior to reaching the sewage treatment stations. This may account for the high concentrations of sewage pollutants in villages and towns in northern Shaanxi.

Central Shaanxi is located in the plain area, where soil and hydrological conditions are relatively better. The quality of the planning and construction of the sewerage pipe networks in the area is high, thus the mixing of other sewage and surface drainage into the pipes is rare. The concentration of pollutants in the sewage of villages and towns entering treatment stations in this region is consistent with that of pollutants in typical rural sewag^2^.

Southern Shaanxi is located in a mountainous area with numerous river systems, abundant rainfall and water resources. Due to the less developed regional economy and the serious limitations caused by the topography of the region, the construction of rural sewage pipelines in this region has lagged behind others, and hence the water quality of the treatment station influent was low, with surface water often imported into the pipelines. The dilution by rainwater and surface water is the main reason for the low pollutant concentrations in rural sewage in southern Shaanxi.

The B/D (BOD_5_/COD), C/N (COD/TN) and C/P (COD/TP) ratios of rural sewage in Shaanxi are presented in Table 1. The B/D of rural sewage in Shaanxi were around 0.40, which suggest that rural sewage in Shaanxi has good biochemical characteristics, and is therefore appropriate for biological wastewater treatment^20^. The C/N and C/P ratios in Shaanxi were lower than the theoretical values required for nutrient (nitrogen and phosphorus) removal in BNR. For a good performance of nitrogen removal, wastewater COD/TN ration higher than 10 were recommended^21,22^. Nowadays domestic wastewater in many rural districts of China has low C/N ratio, sometimes lower than 5^23^, resulting in poor total nitrogen removal (below 50%) due to the lack of sufficient carbon sources in the denitrification process. Rural sewage in Shaanxi is characterized by low carbon source content, which has a serious negative impact on the efficient operation of BNR system.

**Table 1:** The BOD_5_/COD, COD/TN and COD/TP ratios in rural sewage in Shaanxi Province, China.

Item	Northern Shaanxi	Central Shaanxi	Southern Shaanxi
BOD_5_/COD	0.40	0.38	0.41
COD/TN	5.42	5.24	4.23
COD/TP	48.99	49.03	49.37

### Study on fluctuation and discontinuity characteristic of rural sewage

**Figure 2:**
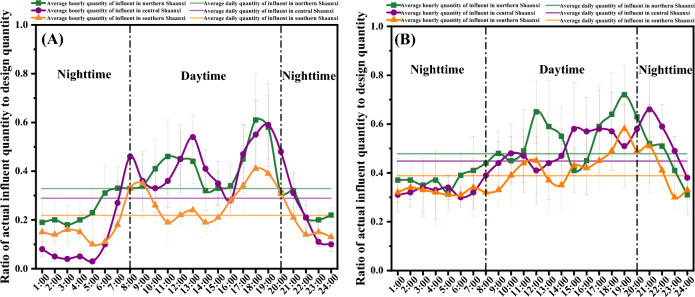
Daily influent fluctuation characterization of village (**A**) and town (**B**) in Shaanxi province, China.

The daily fluctuation characterization of rural sewage for northern, central and southern regions in Shaanxi province, China is shown in Fig. 2. The daily inflow quantity of the village sewage treatment stations (20–200 tons/day) has an obvious fluctuation rhythm (Fig. 2A), with the peak inflow concentrated in three time periods: 08:00 in the morning, 13:00 at midday and 18:00 in the afternoon. The average daily influent loads of village sewage treatment stations in northern Shaanxi, central Shaanxi and southern Shaanxi are 33%, 29% and 22%, respectively. As shown in Fig. 2B, the daily inflow quantity of the town sewage treatment stations (240–2000 tons/day) has an obvious fluctuation as well, with the peak inflow also concentrated in the same three time periods. The average daily influent quantity load of town sewage treatment stations in northern Shaanxi, central Shaanxi and southern Shaanxi are 48%, 45% and 39%, respectively. Compared with village sewage treatment station peak flow times, the peak inflows of town sewage treatment stations have obvious lag times, which is most likely related to long sewage transportation distances in towns compared to those in villages.

**Figure 3:**
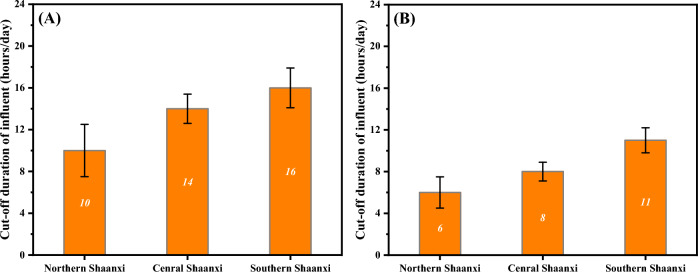
Cut-off durations of rural sewage treatment stations in villages (**A**) and towns (**B**) for Shaanxi province, China.

Cut-off durations of rural sewage treatment stations were investigated in Fig. 3, which shows the cut-off duration in 1 day of rural sewage for Shaanxi. The average cut-off durations of village sewage treatment plants in northern, central and southern Shaanxi were 10, 14 and 16 h/day, respectively (Fig. 3A). The average cut-off durations for town sewage treatment plants in northern, central and southern Shaanxi were 6, 8 and 11 h/day, respectively (Fig. 3B). Station cut-offs were mostly concentrated at night (8 pm to 8am) for both village and town stations. Hence most rural sewage treatment stations were in a condition of discontinuous influent, which would cause a huge obstacle to the stable operation of BNR system.

Above all, the quality of rural sewage in Shaanxi Province, China has good biodegradability, and the B/D value is 0.38–0.41. But the carbon source in sewage is insufficient for nutrient removal in BNR, the values of C/N and C/P are 4.23–5.42 and 48.99–49.37, respectively. Rural sewage in Shaanxi Province, China has obvious fluctuating signatures, mainly manifested as low quantity and discontinuous discharge. The amount of sewage cannot reach 50% of the designed capacity of sewage treatment facilities, and the range of cut-off duration of sewage was 6–16 h/day. Such sewage signatures will bring great obstacles to the stable operation of rural sewage treatment facilities.

### Study on pollutant removal performance of rural sewage treatment stations

**Figure 4:**
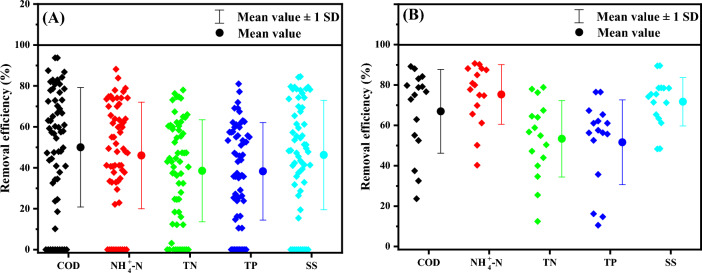
Pollutants removal efficiency of village (**A**) and town (**B**) wastewater treatment stations for Shaanxi province, China.

According to the design scale, the 63 village and town sewage treatment stations sampled in this study can be divided into two sections: < 200 tons/day (village-level) and 240 to 2000 tons/day (town-level) respectively. The nutrient removal rate of each village and town sewage treatment station are shown in Fig. 4. It can be seen that the average removal rates of COD, NH_4_^+^-N, TN, TP and SS at the village-level sewage treatment stations were 50.0 ± 29.2%, 46.0 ± 26.1%, 38.5 ± 24.9%, 38.3 ± 23.8% and 47.1 ± 26.9%, respectively (Fig. 4A), and the corresponding values at the town-level sewage treatment stations were 67.0 ± 20.7%, 75.3 ± 14.7%, 53.4 ± 18.9%, 51.6 ± 21.0% and 71.7 ± 12.0%, respectively (Fig. 4B). The removal efficiency of pollutants in rural sewage treatment stations with different processes is shown in Fig. 2 of the Supplementary Material.

Compared with village-level sewage treatment stations, town-level sewage treatment stations had better pollutant removal efficiencies, which is most likely related to the difference in operation and maintenance modes between village-level and town-level sewage treatment stations. Town-level sewage treatment stations usually employ operation and maintenance personnel on-site, which can deal with abnormal situations in the daily operation process in a timely way. Village-level sewage treatment stations usually adopt operation and maintenance modes using regular inspection by off-site staff, thus making it difficult to deal with abnormal situations in time, reducing the energy efficiency of pollutant removal.

The A^2^/O and MBR processes were the main processes in the sampling survey of rural sewage treatment stations in this study. These two mainstream activated sludge wastewater treatment processes can usually remove more than 80% to 90% of conventional pollutants under normal influent conditions^24–26^. Figure 4 shows the pollutant removal efficiencies for the rural sewage treatment stations observed in this study. The average removal efficiency of conventional pollutants in the surveyed rural sewage treatment stations was only between 38 ± 24% and 75 ± 15%. This was probably due to the intermittent nature of sewage flow in rural areas.

**Figure 5:**
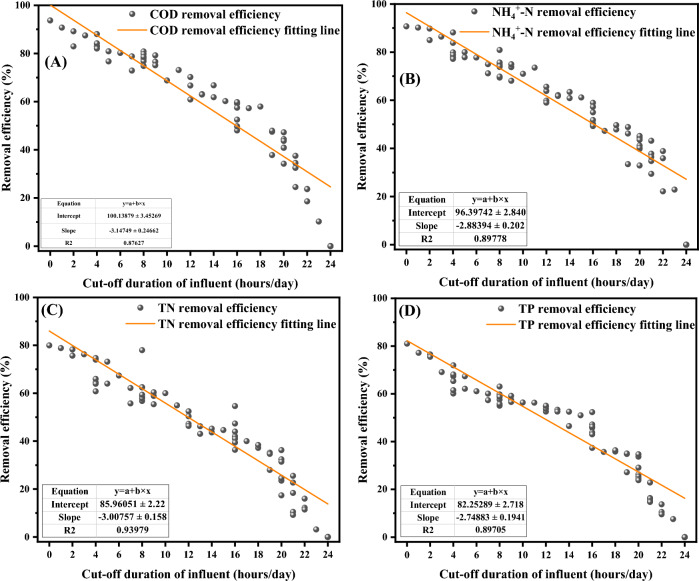
Relationship between nutrient removal rate and the cut-off duration.

The relationship between nutrient removal rate of rural wastewater treatment stations and the cut-off duration is shown in Fig. 5. The results show that the removal rates of COD (Fig. 5A), NH_4_^+^-N (Fig. 5B), TN (Fig. 5C) and TP (Fig. 5D) at the 63 rural sewage treatment stations showed a negative correlation with the cut-off durations, the removal rates of all four pollutant indexes all decreased with increasing cut-off duration.

The direct impact of influent discontinuous on BNR system is that the substrate which microorganisms depend on for survival is insufficient, which makes the functional bacteria in a state of starvation for a long time. Normally under this condition, bacteria become less active by tweaking their metabolism to reduce their need for energy. As an independent mechanism, the bacteria may also switch on programmed cell death (PCD), a genetically programmed process of cell self-destruction that is triggered only under extreme starvation conditions^27^. The main purpose of this process is maintaining partial population activity to avoid losing an advantage in competition with other microbial populations, and this process will lead to cell death^27^.

The direct impact to a BNR system brought by either the activity reduction of functional bacteria or cell death is the reduction of the pollutants removal efficiency, which is the main internal reason why sewage treatment stations using this process cannot operate normally under conditions of intermittent sewage inflow from villages and towns.

### Challenges and recommendations

According to the results of rural sewage discharge characteristics in Shaanxi Province, China in this study, the following challenges need to be considered in order to optimize rural sewage treatment:

(1) Rural sewage usually only contains domestic sewage with low concentration of organic pollutants, thus the concentration of BOD_5_ is low, which leads to insufficient carbon in the influent of sewage treatment facilities. This will seriously reduce the efficiency of nitrogen and phosphorus removal in BNR systems. (2) Since residents in rural areas use little water and usually only produce sewage at specific times of the day, the rural sewage treatment station operated without influent for most of the time. This has a huge impact on the stable operation of BNR systems.

The instability of hydraulic and organic loads has brought great obstacles to the stable operation of rural sewage treatment facilities. The conventional way to solve the fluctuation of rural sewage quantity is to set up regulating ponds. But the regulating tank can not effectively solve the problem of insufficient hydraulic load. The insufficient organic load can be supplemented with additives (glucose, starch, acetate, etc.) to increase the carbon source^28^. The procurement cost of these additives is up to 70% of the operating cost of the entire wastewater treatment plant^29^. This will put a huge economic burden on the already underdeveloped rural areas. Supplementing carbon sources with additives is not a long-term solution.

In view of the above challenges, this paper constructed the BEICT system: the sewage treated by BNR systems can be mixed with sludge fermentation broth in specified proportions. The mixed liquor can be stored and used during influent discontinuation periods, so that the BNR system can undergo continuous operation using internal circulation. Figure 8 suggests the BEICT mode for rural sewage, part of the tail water produced by the bioreactor during the daytime is collected into the mixing tank. The excess sludge discharged by the bioreactor is anaerobic fermented. The VFAs in the fermentation tank are accumulated through process regulation, and the supernatant of the fermentation liquid is discharged into the mixing tank and mixed with the tail water. The mixed liquor contains a large amount of VFAs, so it is more conducive to the growth and metabolism of microorganisms in the BNR system. When the carbon source of the bioreactor is insufficient or there is no wastewater treatment at nighttime, the mixed liquor in the mixing tank can be recycled to realize the supplement of carbon and water source.

### Operation effect of the BEICT bioreactor

**Figure 6:**
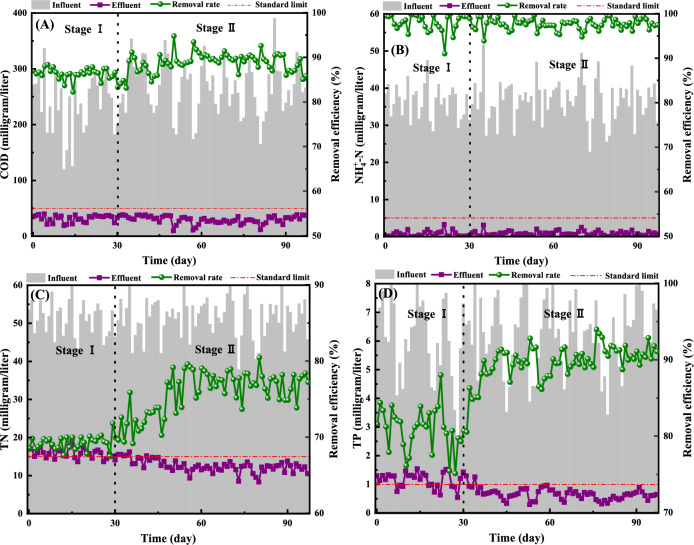
Removal performance of COD (**A**), NH_4_^+^-N (**B**), TN (**C**) and TP (**D**) in each stage.

The pollutant removal efficiency of the bioreactor is shown in Fig. 6. As can be seen from Fig. 6A and B, there is little difference in the removal effect of the system on COD and NH_4_^+^-N in stage I and II. The average removal rates of COD and NH_4_^+^-N in the whole experiment were 88.42% and 96.55%, respectively. The corresponding average effluent concentrations were 31.24 and 0.74 mg/L, respectively. The results showed that the influent mode of the mixture of recharge tail water and sludge fermentation liquid during cut-off period did not affect the removal performance of COD and NH_4_^+^-N in the BEICT system. In the whole process of the experiment, the BEICT system maintained an efficient and stable removal efficiency of COD and NH_4_^+^-N, and the corresponding average effluent concentration could meet the national water quality standard limits.

As can be seen from Fig. 6C and D, the removal efficiency of TN and TP in stage II showed an obvious increasing trend compared with stage I. The average TN removal rates of the system in stage I and II were 68.11% and 77.42%, respectively, and the corresponding average effluent concentration was 16.14 and 12.46 mg/L. The average TP removal rates of the system in stage I and II were 79.63% and 89.69%, respectively, and the corresponding average effluent concentration was 1.14 and 0.65 mg/L. The results showed that the influent mode of the mixture of recharge tail water and sludge fermentation liquid during cut-off period can improve the removal performance of TN and TP in BEICT system.

Hu et al.^18^ successfully increased the removal rates of NH_4_^+^-N and PO_4_^3+^-P to 92.38% and 92.70% by adding food waste fermentation liquid to the batch reactor (SBR). The addition of fermentation liquid of food waste increased the components of VFAs and PHAs in the influent, which was the main reason for the improvement of nitrogen and phosphorus removal efficiency of the system. Liu et al.^19^ successfully coupled the sludge anaerobic fermentation unit with the sewage treatment system in an urban sewage treatment plant, achieving a sludge reduction rate of 54.19%, and increasing the system's removal rates of TN and TP to 72.39% and 89.65%. The above studies are based on stable influent conditions. In this study, the BEICT system achieved stable and efficient nitrogen and phosphorus removal efficiency through the operation mode of mixed liquid internal circulation under the condition of influent interruption for 12 h per day. The results showed that the BEICT system can adapt to the discontinuous discharge characteristics of sewage in rural areas. The mechanism of nitrogen and phosphorus removal, sludge reduction effect and economic benefits of the BEICT system are worthy of further study.

**Figure 7:**
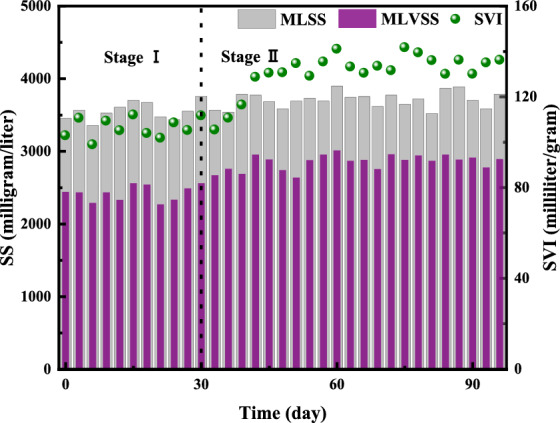
Characteristics of activated sludge in each stage.

The variation of MLSS, MLVSS and SVI of activated sludge in the aerobic zone of the BEICT system are shown in Fig. 7. It can be seen that the activated sludge concentration in the system was relatively stable, and the average MLSS, MLVSS and SVI of the activated sludge in the whole experiment process were 3702 mg/L mg/L, 2943 mg/L and 124 mL/g, respectively. The activated sludge in the system maintained good activity and sludge settling performance, and there was no sludge bulking phenomenon. The average MLVSS/MLSS of stage I and II were 0.63 and 0.72, respectively, and the average MLVSS/MLSS of activated sludge in the system was increased in stage II, which further indicated that the sludge activity was improved in stage II. The SVI of activated sludge in the system increased in stage II, and the average SVI of stage I and II were 104 and 128 mL/g, respectively. The influent mode of the mixture of recharge tail water and sludge fermentation liquid during cut-off period will worsen the sedimentation performance of activated sludge, but it will not adversely affect the sludge activity and pollutant removal performance of the system.

## Conclusion

This study found that rural sewage in Shaanxi Province, China has significant discontinuous discharge characteristics, and the range of cut-off duration of sewage was 6–16 h/day, which leads to poor pollutant removal efficiency (COD: 50.0 ± 29.2%, NH_4_^+^-N: 46.0 ± 26.1%, TN: 38.5 ± 24.9% and TP: 38.3 ± 23.8%) of rural sewage treatment stations. A pilot-scale of rural sewage BEICT system was constructed. Long-term operation indicated the adoption of this system could increase TN and TP removal rates to 72.39% and 89.65% under the condition of influent interruption for 12 h per day. Moreover, the activated sludge of BEICT system could maintained good activity and stable sedimentation performance, with MLVSS/MLSS and SVI of 0.72 and 128 mL/g, respectively. The results showed that the BEICT system can adapt to the discontinuous discharge characteristics of sewage in rural areas, and has important significance for guiding the treatment of rural domestic sewage in China.

## Methods

### Survey methodology

According to the topographic characteristics of Shaanxi Province, this study investigated 15 rural sewage treatment stations in northern Shaanxi, 16 rural sewage treatment stations in central Shaanxi and 32 rural sewage treatment stations in southern Shaanxi. The treatment processes of all these sewage treatment stations included A^2^/O, MBR, CWs and oxidation pond. The survey took place from June 2021 to June 2022. Basic information describing all 63 sewage treatment stations are presented in the Supplementary Material. Field investigation is mainly carried out in the form of field investigation, water sample collection, water quality analysis and information collection. The construction units, operation units and relevant government departments of local sewage treatment facilities participated in or cooperated with the research work. The questionnaire of this study can be seen in the Supplementary Material The influent quantity of each rural sewage treatment station was obtained by the on-site staff regularly observing the flow meter. The influent and effluent samples from each sample site will be taken 100 mL at 7:00 A.M., 12:00 P.M. and 19:00 P.M. respectively and mixed. Sampling was not less than 4 times a week, and there is no rain before each sampling. The mixed samples were transported to the laboratory in refrigerated storage at 3 °C and were analyzed within 3 days. The main analysis items in this study include COD, BOD_5_, NH_4_^+^-N, TN, TP and SS. These indexes were measured by standard methods^30^.

### The BEICT pilot-scale bioreactor

**Figure 8:**
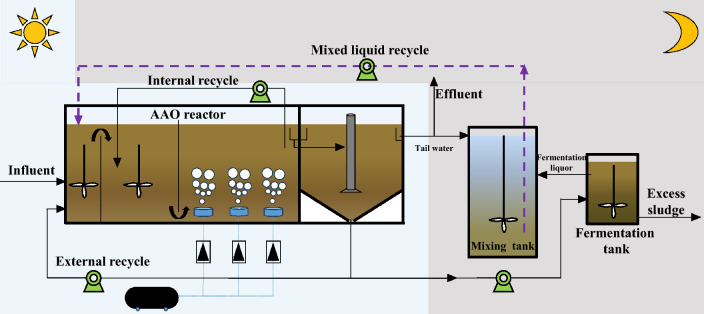
Schematic diagram of rural sewage BEICT bioreactor.

The plane diagram of the BEICT pilot-scale bioreactor is shown in Fig. 8. The effective volume of the bioreactor is 6.8 m^3^, in which the effective volume ratio of anoxic zone, anoxic zone and aerobic zone is 1:3:4. The anaerobic and anoxic zones in the bioreactor are equipped with mechanical stirring devices. The concentration of dissolved oxygen (DO) in the aerobic pool was controlled at 2.0–3.0 mg/L by rotameter. The influent was controlled by time relay to simulate the condition of rural sewage discontinuous discharge. During the whole experiment, the influent flow rate of the system was controlled at 0.5 ± 0.05 tons/h, and the temperature in the bioreactor was maintained at 18 ± 2 °C.

The experiment in this study was conducted for a total of 97 days and was divided into 2 phases. In stage I (0–30 day), the influent mode of the system was the traditional continuous flow, the main purpose of which is to compare with the experimental results of the next stage. In stage II (31–97 day), the influent mode of the system was adjusted to discontinuous flow, the bioreactor operated with influent for 12 h (8:00–20:00) and without influent for 12 h (20:00–8:00). The organic and hydraulic load of the system was maintained by replenishment of the mixture of tail water and fermentation liquid from mixing tank during the influent cut-off period at night. The main purpose of the second stage is to study whether the bioreactor can maintain its pollutant removal performance by replenishing the tail water and sludge fermentation liquid mixture under the condition of long-term intermittent discontinuous flow. Other operating parameters of the bioreactor are shown in Table 2.

**Table 2:** Operational conditions for this study.

Stage	Time (day)	Influent mode	SRT (day)	HRT (hours)	Internal recycle	External recycle	Mixed liquid recycle	MLSS (mg/L)
I	0–30	Continuous	13 ± 2	14	200%	100%	100%	3714 ± 122
II	31–97	Discontinuous (cut-off duration = 12 h/day)						

### Wastewater and sludge

The influent of the bioreactor is the effluent from the regulation tank of a rural wastewater treatment plant (WWTP) in southern Shaanxi Province. The scale of the WWTP is 1000 tons/day and the traditional A^2^/O process is adopted. The pollutants concentration of influent is shown in Table 3. The activated sludge of the bioreactor was obtained from the aerobic plant of the WWTP, and the sludge maintained high activity. After 30 days of acclimation and adaptation, the removal performance of pollutants in the bioreactor tended to be stable.

**Table 3:** Influent characteristics of the bioreactor.

Item	pH	COD/(mg/L)	TN (mg/L)	TP (mg/L)	NH_4_^+^-N (mg/L)
Range	7.24–7.83	115.26–371.16	35.24–64.77	2.17–7.91	24.30–52.26
Mean value	7.51	251.65	50.25	5.87	35.42

### Preparation of fermentation liquid

The effective volume of the fermenter was 2 cubic meters. The excess sludge (1.0 tons) of the bioreactor was used as the inoculated sludge and acclimated at 30 °C (without adjusting pH). After 10–20 days of acclimation, the fermenter basically tended to a stable state. From the 31 d of the experiment, the bioreactor discharged 0.4–0.5 tons of excess sludge into the fermenter for anaerobic fermentation every day, and then extracted 0.4–0.5 tons of fermentation liquid from the fermenter into the mixing tank for mixing with tail water. The mixing ratio of tail water and fermentation liquid was 12:1. The composition and properties of fermentation liquid and mixture are shown in Table 4.

**Table 4:** Influent characteristics of the bioreactor.

Parameter	Fermentation liquid	Mixed liquid (12:1)
pH	5.33 ± 0.15	6.22 ± 0.24
MLSS (mg/L)	18,246 ± 1775	1637 ± 242
COD (mg/L)	5335.21 ± 432.14	511.26 ± 133.18
SCOD (mg/L)	1159.66 ± 177.22	311.28 ± 42.33
VFAs (mg/L)	507.15 ± 112.66	55.43 ± 5.88
Acetic acid (mg/L)	277.37 ± 24.65	22.14 ± 2.37
Propionic acid (mg/L)	42.65 ± 5.77	5.55 ± 0.43
Protein (mg/L)	572.25 ± 87.46	62.11 ± 4.47
Carbohydrate (mg/L)	321.22 ± 54.15	32.37 ± 2.28
TN (mg/L)	662.14 ± 123.56	88.11 ± 8.98
TP (mg/L)	81.25 ± 7.73	16.87 ± 4.55

### Experimental methods

Sludge volume index (SVI), mixed liquid suspended solids concentration (MLSS), mixed liquid volatile suspended solids concentration (MLVSS), COD, NH_4_^+^-N, TN and TP concentrations were determined by standard methods^30^. DO, pH and water temperature are monitored using Hashi's WTW multifunction automatic tester. The determination methods of VFAs, acetic acid and propionic acid are described in the previous study^31^. Protein was determined by LOWRY method^32^. Carbohydrate was determined by phenol–sulfuric acid method with glucose as the base material^32^.

### Supplementary Information

Supplementary Information.

## Data Availability

All data generated or analyzed during this study are included in this published article.
