# Supplementary material for: Study on sewage characteristics in rural China and pollutants removal performance of biologically enhanced internal circulation treatment system
Source: Sci Rep. 2023 Oct 23;13:18058. doi: 10.1038/s41598-023-45085-4 (PMC10593821; doi:10.1038/s41598-023-45085-4)
Supplement: Supplementary file 1 — Supplementary Information. [file 41598_2023_45085_MOESM1_ESM.docx]

Supplementary Material

Study on sewage characteristics in rural China and pollutants removal performance of biologically enhanced internal circulation treatment system

Yi Rong^*^, Hongjun Tang, Yang Zhang, Yingying Sun, Zhe Liu

*** Correspondence:** Yi Rong: rongyi@xauat.edu.cn

# Supplementary Figures and Tables

## Supplementary Figures

**Supplementary Figure 1.** Rural sewage treatment station information statistics and the flow diagram of the typical rural treatment stations. (A): Statistics by region; (B): Statistics by scale; (C): Statistics by process; (D): Statistics according to management mode; (E) Flow diagram

The rural sewage treatment stations used in this study were selected in order to be representative of the different topographic characteristics of Shaanxi Province, i.e.: 15 treatment stations in the Loess Plateau region of northern Shaanxi Province; 16 treatment stations in the plain region of central Shaanxi Province; and 32 treatment stations in the mountainous region of southern Shaanxi Province, as shown in Supplementary Figure 1 (A).

According to the design scale, the 63 villages and towns sewage treatment stations sampled in this study can be divided into two sections: <200 t/d (village-level) and 240 t/d to 2000 t/d (town-level) respectively, as shown in Supplementary Figure 1 (B).

The village and town sewage treatment stations in this study include 27 A^2^/O sewage treatment stations, 24 MBR sewage treatment stations, 7 constructed wetland (CWs) sewage treatment stations, 4 pretreatment + oxidation pond sewage treatment stations, and 1 CASS sewage treatment station, as shown in Supplementary Figure 1 (C).

The management modes of the rural sewage treatment stations in this study include government management, entrusted the third party and unattended, as shown in Supplementary Figure 1 (D).

Shaanxi rural sewage treatment station is mainly composed of three parts: sewage collection system, treatment system and discharge system. The black water in most rural areas is collected and treated by septic tanks and cleaned regularly for fertilization. In some villages with better pipe network, black water usually enters the sewage network after collection and treatment by septic tank. The process of sewage treatment station is mainly A^2^/O, followed by MBR. The advanced treatment process is mainly based on constructed wetland, which is quite common in southern Shaanxi. The Flow diagram is shown in Supplementary Figure 1 (E).

**Supplementary Figure 2.** Removal efficiency of COD (A), NH_4_^+^-N (B), TN (C) and TP (D) in rural sewage treatment stations with different process

As can be seen from Supplementary Figure 2, the average COD removal rates of rural sewage treatment stations with A^2^/O, MBR, constructed wetland and oxidized pond processes were 69.6±15.5, 47.1±31.3, 28.6±27.0 and 38.7±30.3%, respectively. The average removal rates of NH_4_^+^-N were 70.7±13.8, 44.7±26.7, 18.1±17.5 and 30.5±20.3%, respectively. The average removal rates of TN were 61.5±11.7, 33.0±22.9, 11.3±15.9 and 18.7±12.4%, respectively. The average removal rates of TP were 59.5±9.6, 32.2±23.1, 32.3±30.7 and 14.7±10.8%, respectively. The results showed that A^2^/O has better pollutant removal efficiency than MBR, constructed wetland and oxidized pond processes, which reflected the better adaptability of A^2^/O process in rural areas.

Although constructed wetland technology has many advantages, it is more susceptible to the influence of climate and environment, so there are some drawbacks in the engineering application of rural sewage treatment, and the impact resistance performance is weak. Constructed wetlands often need to combine with other water treatment technology, play a role together, and then achieve better treatment effect. Constructed wetlands are often used in rural areas for deep treatment at the end of wastewater treatment system and play the functions of water storage and landscape creation.

## Supplementary Table

- **Supplementary Table 1.** Basic information of 63 sewage treatment stations

| No. | Sewage treatment stations | Scale (m^3^/d) | Main unit | Management model | Area |
| --- | --- | --- | --- | --- | --- |
| 1 | Nangou village sewage treatment station | 50 | MBR | Entrust the third party | Ansai, northern Shaanxi |
| 2 | Louping village sewage treatment station | 50 | MBR | Entrust the third party | Ansai, northern Shaanxi |
| 3 | Gaoqiao village sewage treatment station | 50 | MBR | Entrust the third party | Ansai, northern Shaanxi |
| 4 | Hougoumen village sewage treatment station | 50 | MBR | Entrust the third party | Ansai, northern Shaanxi |
| 5 | Gaogoumen village sewage treatment station | 50 | MBR | Entrust the third party | Ansai, northern Shaanxi |
| 6 | Wangyao village sewage treatment station | 50 | MBR | Entrust the third party | Ansai, northern Shaanxi |
| 7 | Huaziping town Ⅱ sewage treatment station | 50 | MBR | Entrust the third party | Ansai, northern Shaanxi |
| 8 | Liandaowan village sewage treatment station | 50 | MBR | Entrust the third party | Ansai, northern Shaanxi |
| 9 | Pingqiao sewage treatment station | 50 | MBR | Entrust the third party | Ansai, northern Shaanxi |
| 10 | Wangjiawan village sewage treatment station | 50 | MBR | Entrust the third party | Ansai, northern Shaanxi |
| 11 | Jianhua Temple sewage treatment station | 200 | MBR | Entrust the third party | Ansai, northern Shaanxi |
| 12 | Zhuanyaowan town sewage treatment station | 240 | A^2^/O | Entrust the third party | Ansai, northern Shaanxi |
| 13 | Xihekou town sewage treatment station | 250 | MBR | Entrust the third party | Ansai, northern Shaanxi |
| 14 | Huaziping town Ⅰ sewage treatment station | 400 | MBR | Entrust the third party | Ansai, northern Shaanxi |
| 15 | Zhaoan town sewage treatment station | 500 | A^2^/O | Entrust the third party | Ansai, northern Shaanxi |
| 16 | Sheshu village sewage treatment station | 30 | Pretreatment + Oxidation pond | Unattended | Jingyang, central Shaanxi |
| 17 | Qujia village sewage treatment station | 30 | Pretreatment + Oxidation pond | Unattended | Jingyang, central Shaanxi |
| 18 | Baizhang village sewage treatment station | 30 | Pretreatment + Oxidation pond | Unattended | Jingyang, central Shaanxi |
| 19 | Liumeng village sewage treatment station | 30 | Pretreatment + Oxidation pond | Unattended | Jingyang, central Shaanxi |
| 20 | Guanmiao village sewage treatment station | 40 | Constructed wetland | Unattended | Jingyang, central Shaanxi |
| 21 | Beizhao village sewage treatment station | 75 | Constructed wetland | Unattended | Jingyang, central Shaanxi |
| 22 | Wangqiao town sewage treatment station | 500 | A^2^/O | Government management | Jingyang, central Shaanxi |
| 23 | Hujiawan village sewage treatment station | 30 | Constructed wetland | Unattended | Binzhou, central Shaanxi |
| 24 | Lu village sewage treatment station | 80 | Constructed wetland | Unattended | Binzhou, central Shaanxi |
| 25 | Yongle village sewage treatment station | 100 | Constructed wetland | Unattended | Binzhou, central Shaanxi |
| 26 | Yaochitou village sewage treatment station | 120 | Constructed wetland | Unattended | Binzhou, central Shaanxi |
| 27 | Gao village sewage treatment station | 200 | Constructed wetland | Unattended | Binzhou, central Shaanxi |
| 28 | Xinmin town sewage treatment plant | 2000 | CASS | Government management | Binzhou, central Shaanxi |
| 29 | Beiji town sewage treatment plant | 2000 | MBR | Government management | Binzhou, central Shaanxi |
| 30 | Miaojiapo village sewage treatment station | 30 | A^2^/O | Government management | Lantian, central Shaanxi |
| 31 | Baoxing Temple village sewage treatment station | 50 | A^2^/O | Government management | Lantian, central Shaanxi |
| 32 | Qiaoshan Zuoan sewage treatment station in Shuanglong town | 20 | A^2^/O+Constructed wetland | Entrust the third party | Hanbin, southern Shaanxi |
| 33 | Qiaoshan Yuoan sewage treatment station in Shuanglong town | 20 | A^2^/O+Constructed wetland | Entrust the third party | Hanbin, southern Shaanxi |
| 34 | Hejiatang sewage treatment station in Huangni Village | 20 | A^2^/O+Constructed wetland | Entrust the third party | Hanbin, southern Shaanxi |
| 35 | Guandimiao sewage treatment station in Huangni Village | 20 | MBR | Entrust the third party | Hanbin, southern Shaanxi |
| 36 | Erlian Ⅴ sewage treatment station in Dazhuyuan town | 20 | A^2^/O+Constructed wetland | Entrust the third party | Hanbin, southern Shaanxi |
| 37 | Xipo primary school sewage treatment station in Yinghu town | 30 | A^2^/O+Constructed wetland | Entrust the third party | Hanbin, southern Shaanxi |
| 38 | Shagou primary school sewage treatment station in Yinghu town | 30 | A^2^/O+Constructed wetland | Entrust the third party | Hanbin, southern Shaanxi |
| 39 | Xieping sewage treatment station in Shuanglong town | 30 | A^2^/O+Constructed wetland | Entrust the third party | Hanbin, southern Shaanxi |
| 40 | Yuanba sewage treatment station in Hongshan town | 30 | A^2^/O+Constructed wetland | Entrust the third party | Hanbin, southern Shaanxi |
| 41 | Lijiaqiao sewage treatment station in Huangni Village | 30 | A^2^/O+Constructed wetland | Entrust the third party | Hanbin, southern Shaanxi |
| 42 | Xinghuo sewage treatment station in Liushui town | 30 | A^2^/O+Constructed wetland | Entrust the third party | Hanbin, southern Shaanxi |
| 43 | Erlian Ⅳ sewage treatment station in Dazhuyuan town | 40 | MBR | Entrust the third party | Hanbin, southern Shaanxi |
| 44 | Xinxing sewage treatment station in Yinghu town | 50 | MBR | Entrust the third party | Hanbin, southern Shaanxi |
| 45 | Guojiahe sewage treatment station in Yinghu town | 50 | MBR | Entrust the third party | Hanbin, southern Shaanxi |
| 46 | Guihua sewage treatment station in Yinghu town | 50 | MBR | Entrust the third party | Hanbin, southern Shaanxi |
| 47 | Yuejin sewage treatment station in Yinghu town | 50 | A^2^/O+Constructed wetland | Entrust the third party | Hanbin, southern Shaanxi |
| 48 | Longquan Ⅶ sewage treatment station in Shuanglong town | 50 | MBR | Entrust the third party | Hanbin, southern Shaanxi |
| 49 | Qianlongsewage treatment station in Hongshan town | 50 | A^2^/O+Constructed wetland | Entrust the third party | Hanbin, southern Shaanxi |
| 50 | Shuangbai sewage treatment station in Hongshan town | 50 | MBR | Entrust the third party | Hanbin, southern Shaanxi |
| 51 | Hexin sewage treatment station in Liushui town | 50 | A^2^/O+Constructed wetland | Entrust the third party | Hanbin, southern Shaanxi |
| 52 | Fenghuang sewage treatment station in Liushui town | 50 | A^2^/O+Constructed wetland | Entrust the third party | Hanbin, southern Shaanxi |
| 53 | Dazhuyuan village Ⅸ sewage treatment station | 50 | MBR | Entrust the third party | Hanbin, southern Shaanxi |
| 54 | Xipo sewage treatment station in Yinghu town | 80 | A^2^/O+Constructed wetland | Entrust the third party | Hanbin, southern Shaanxi |
| 55 | Chazhai sewage treatment station in Dazhuyuan town | 150 | A^2^/O+Constructed wetland | Entrust the third party | Hanbin, southern Shaanxi |
| 56 | Xinba sewage treatment plant in Liushui town | 400 | A^2^/O+Cloth filter | Entrust the third party | Hanbin, southern Shaanxi |
| 57 | Jiangnan sewage treatment in Yinghu town | 500 | MBR | Entrust the third party | Hanbin, southern Shaanxi |
| 58 | Jiangbei sewage treatment in Yinghu town | 500 | MBR | Entrust the third party | Hanbin, southern Shaanxi |
| 59 | Shuanglong town sewage treatment plant | 500 | A^2^/O+Constructed wetland | Entrust the third party | Hanbin, southern Shaanxi |
| 60 | Hongshan Town Sewage Treatment Plant | 500 | A^2^/O+Constructed wetland | Entrust the third party | Hanbin, southern Shaanxi |
| 61 | Liushui town sewage treatment plant | 800 | A^2^/O+ Cloth filter | Entrust the third party | Hanbin, southern Shaanxi |
| 62 | Dazhuyuan sewage treatment plant | 800 | A^2^/O+Constructed wetland | Entrust the third party | Hanbin, southern Shaanxi |
| 63 | Qiyan sewage treatment plant in Dazhuyuan town | 1000 | A^2^/O+Sand filter | Entrust the third party | Hanbin, southern Shaanxi |

- **Supplementary Table 2.** Concentration of influent pollutants of rural sewage treatment plants in various regions

| District | COD/ (mg·L^-1^) | BOD_5_/ (mg·L^-1^) | TN/ (mg·L^-1^) | TP/ (mg·L^-1^) |
| --- | --- | --- | --- | --- |
| This study | 100~213 | 38~86 | 24~39 | 2~4 |
| Beijing | 105~445 | 34~105 | 21~55 | 1.4~5.8 |
| Jiangsu | 120~400 | 80~200 | 70~80 | 4.5~6.5 |
| Yunnan | 30~300 | 11~150 | 20~30 | 4.2~15 |
| Sichuan | 60~120 | 40~70 | 40~70 | 0.5~2.1 |
| Guangdong | 182~350 | 80~300 | 50~100 | 1.9~2.3 |
| Zhejiang | 135~639 | 63~398 | 37~76 | 2.1~9.1 |

As can be seen from the **Table 2**, there is an obvious difference between the concentration of pollutants in rural sewage in Shaanxi province and that in Jiangsu, Guangdong and other economically developed areas in China, which is mainly related to the relatively backward network construction and residents' living habits in rural Shaanxi Province.

- **Supplementary Table 3.** Basic information questionnaire

| **Regional overview** | | **Overview of WWTP** | | **Sewage source** | | | **Operational aspect** | | **Drainage** | | **Pollutants concentration of influent and effluent** | | | | | | | | |
| --- | --- | --- | --- | --- | --- | --- | --- | --- | --- | --- | --- | --- | --- | --- | --- | --- | --- | --- | --- |
|  |  |  |  |  |  |  |  |  |  |  | **Index** | **Design influent quality** | **Effluent quality standard** | **Influent quality in the last 3 days** | | | **Effluent quality in the last 3 days** | | |
|  |  |  |  |  |  |  |  |  |  |  |  |  |  | **Day 1** | **Day 2** | **Day 3** | **Day 1** | **Day 2** | **Day 3** |
| **Name of WWTP** |  | **Process and scale** |  | **Actual scale** |  | | **Operation or not** |  | **Drainage direction (arable land/river/other)** |  | **COD** |  |  |  |  |  |  |  |  |
| **Jurisdiction (village, town)** |  | **Construction and operation cost** |  | **Sewage Type (Domestic/Industrial/Mixed)** |  | | **Whether there is an operation company** |  |  |  | **NH_4_^+^-N** |  |  |  |  |  |  |  |  |
|  |  | **Construction time** |  | **Sewerage status (pipe network/open channel/culvert/other)** |  | | **Whether there is a work log** |  | **Quantity of effluent** |  | **TN** |  |  |  |  |  |  |  |  |
|  |  | **Population and villages served** |  | **Continuous or not (if not, please investigate the cut-off period)** |  | | **Whether water quality online monitoring is running properly** |  |  |  | **TP** |  |  |  |  |  |  |  |  |
|  |  |  |  |  |  |  |  |  |  |  | **SS** |  |  |  |  |  |  |  |  |
| **On-site problems** | |  | | | | | | | | | | | | | | | | | |
| **Recorder：** | | | | | | **Phone number：** | | | | | | | **Date：** | | | | | | |

- **Supplementary Table 4.** Water quantity questionnaire

| **No:** | | **Name of WWTP:** | | | **Date:** | |
| --- | --- | --- | --- | --- | --- | --- |
| **Time** | **Influent rate**  **m^3^/h** | | **Effluent rate**  **m^3^/h** | **Voltmeter reading**  **kw/h** | | **Accumulated inflow m^3^** |
|  |  | |  |  | |  |
|  |  | |  |  | |  |
|  |  | |  |  | |  |
|  |  | |  |  | |  |
|  |  | |  |  | |  |
|  |  | |  |  | |  |
|  |  | |  |  | |  |
|  |  | |  |  | |  |
|  |  | |  |  | |  |
|  |  | |  |  | |  |
|  |  | |  |  | |  |
|  |  | |  |  | |  |
|  |  | |  |  | |  |
|  |  | |  |  | |  |
|  |  | |  |  | |  |
|  |  | |  |  | |  |
|  |  | |  |  | |  |
|  |  | |  |  | |  |
|  |  | |  |  | |  |
|  |  | |  |  | |  |
|  |  | |  |  | |  |
|  |  | |  |  | |  |
|  |  | |  |  | |  |
|  |  | |  |  | |  |
|  |  | |  |  | |  |
|  |  | |  |  | |  |
|  |  | |  |  | |  |
|  |  | |  |  | |  |
|  |  | |  |  | |  |
|  |  | |  |  | |  |
|  |  | |  |  | |  |
|  |  | |  |  | |  |
|  |  | |  |  | |  |
|  |  | |  |  | |  |
| **Remark column：** | | | | | | |
